# Epidemiological trends in infant mortality related to necrotizing enterocolitis

**DOI:** 10.3389/fped.2026.1825909

**Published:** 2026-05-14

**Authors:** Sowmya Kolluru, Natalie Liao, Kennedy Haase, Olivia Foley, Lillia Cherkasskiy, Abubakar Tauseef

**Affiliations:** 1 Creighton University School of Medicine, Omaha, NE, United States; 2 Department of Family Medicine, Creighton University School of Medicine, Omaha, NE, United States; 3 Department of Medicine, Creighton University School of Medicine, Omaha, NE, United States

**Keywords:** CDC WONDER, gastroenterology, necrotizing enterocolitis, neonatology, pediatrics

## Abstract

**Introduction:**

Necrotizing enterocolitis (NEC) is the leading cause of gastrointestinal morbidity and mortality among infants in the neonatal intensive care unit. With an overall prevalence of 1%, NEC is found in about 11% of infants with very low birthweights (VLBW) and 22% in infants of extremely low birthweights. A disease of prematurity, NEC has a multifactorial pathogenesis involving a combination of feeding regimens, gut microbiome, and birth weight. This study aims to elucidate the demographic factors associated with NEC- mediated infant mortality, with the goal of guiding future research into disease diagnosis and management.

**Methods:**

The CDC WONDER database was queried to collect data on mortality from NEC of infants <1 year old between 1999 and 2023. Data was stratified by sex, race/ethnicity, urbanization status, and census region. Crude mortality rate (CMR) was determined, and Joinpoint analysis was conducted to identify significant changes in mortality trends.

**Results:**

Overall NEC-related CMR decreased slightly between 1999 and 2023. CMR was higher in males than females throughout the study period, though mortality decreased slightly in the male population. When stratified by race, Non-Hispanic (NH) Black or African American patients had the highest overall CMR, followed by Hispanic or Latino and NH White patients, respectively. Regionally, the South had the highest CMR across the four studied census regions, though Southern mortality decreased over the study period. The West reported the lowest mortality from NEC and was the only census region with reported increase in CMR. In both urban and rural areas, mortality initially increased before a subsequent decrease throughout the study period.

**Discussion:**

This study builds on prior research efforts into mortality trends in NEC to highlight the disproportionate mortality burden associated with NEC faced by certain groups, including males, NH Black of African American patients, and patients from the Southern United States. Further research into the implications of different socioeconomic determinants of health and biomarker variability by demographic cohort can guide more effective diagnostic and management strategies.

## 1 Introduction

As a leading cause of morbidity and mortality among infants in the neonatal intensive care unit (NICU), necrotizing enterocolitis (NEC) remains both a significant clinical and diagnostic challenge across the world (1). NEC involves inflammation and subsequent necrosis of the intestines affecting preterm infants and infants of low (<2500 g), very low (<1500 g), or extremely low birth weights (<1000 g) (1–4). NEC has an overall prevalence of 1%, but the prevalence increases to 11% in very low birth weight (VLBW) infants and 22% in extremely low birth weight infants (2). Moreover, once diagnosed with NEC, infants face a mortality rate of about 23.5% (2). NEC accounts for 5% of NICU admissions and often requires surgical management (5). Among patients who survive past infancy, outcomes remain poor, with increased risk of intestinal failure and neurodevelopmental disorders due to the patient’s state of systemic inflammation (2).

Prematurity is a major risk factor for NEC with a multifactorial pathophysiology, involving a combination of factors including feeding status, gut blood flow, birth weight, and gastrointestinal microbiome (6). With feeding, specifically, optimization in premature infants to limit the development of NEC involves TPN, early progression to enteral feeds, increased protein and caloric intake, and the utilization of maternal or donor milk when feasible is crucial (6). Preterm infants, as well as those who experienced intrauterine growth restriction (IUGR), have immature intestines with poor peristalsis, decreased secretion of gastric acid and digestive enzymes, and mucosa that has increased susceptibility to inflammation-mediated damage (2). Additional risk factors for NEC include time spent on mechanical ventilation and history of infections (7).

Despite improvements in outcomes over time, NEC has remained a significant diagnostic challenge (8). NEC presents similarly to both septic ileus and intestinal perforation, making timely diagnosis and accurate management difficult (1, 5). Despite new research showing initial promise from genomics and proteomics, ultrasound remains a mainstay for exclusion of a NEC diagnosis (5, 6). Preliminary treatment of NEC involves bowel rest, gastric decompression, systemic antibiotics, and parenteral nutrition (9). Surgery is reserved for complications, including intestinal perforation (9). Much of the research effort has focused on prevention of NEC, with factors such as avoiding preterm birth, introducing human milk to the neonate earlier, and administering antenatal steroids (5, 6, 9, 10).

Prior studies have independently characterized epidemiological trends in NEC, though they focused on specific regions of the country or only specific demographic variables. One study of New York NICUs found not only increased incidence among babies with birth weights between 750 and 1,000 grams, but also a 1.53 times higher risk of NEC in urban areas than their rural counterparts and increased incidence among NH Black or African American patients compared to their NH White counterparts (11). Several additional studies have identified similar trends in NEC incidence and NEC-associated mortality across racial groups (12–14). In exploring this trend in mortality from NEC, Wolff et al. also noted disparities between states in NEC-associated mortality, but further study is needed to identify variation over time (14). As such, the objective of this study is to build on existing literature to generate a comprehensive understanding of mortality associated with NEC across the US and characterize mortality variation by numerous demographic variables between 1999 and 2023. With the devastating burden and implications of NEC on infants and the numerous risk factors associated with the disease, elucidation of the factors involved in mortality from NEC is vital to guiding future study into management and prevention of the disease.

## 2 Materials and methods

This study used the Center for Disease Control and Prevention Wide-ranging Online Data for Epidemiologic Research (CDC WONDER) to identify mortality related to NEC within the United States (US) (15). Specifically, the Multiple Cause of Death database was queried to analyze deaths of individuals <1 year of age for whom a cause of death was listed under the International Classification of Diseases, 10th Revision, Clinical Modification code (ICD) P77 (Necrotizing enterocolitis of newborn). The CDC WONDER database defines a cause of death as one for which the ICD code is listed on the death certificate. Up to 20 causes of death can be listed. This manuscript will describe mortality rates as “NEC-related mortality,” to indicate the NEC was listed as one of the causes of death on the death certificate. Limitations to identifying primary cause of death in these patients is addressed in the “Limitations” section below. This database was precluded from IRB approval due to its inclusion of publicly available, deidentified information from death certificate records (16).

Data from NEC-related mortality and population size was extracted from the years 1999 to 2023 and reported in the form of raw deaths and crude mortality rate (CMR). Data was additionally stratified according to sex, race/ethnicity, census region, and urbanization status. Race/ethnicity was classified as Hispanic or Latino, NH White, and NH Black or African American. Census Regions were classified into South, West, Midwest, and Northeast according to the Census Bureau definitions (17). Urbanization status was classified according to the 2013 National Center for Health Statistics Urban-Rural Classification Scheme (NCHS) Urban-Rural Classification Scheme for Counties (18). This particular classification divided the population into urban (metropolitan areas with >50,000 individuals) and rural (non-metropolitan with population <50,000) areas. Populations whose death count was less than 20 was deemed to be unreliable by the CDC WONDER database and was excluded from analysis for this manuscript.

This study determined trends in mortality over time using the Joinpoint Regression Program (Joinpoint version 5.4.0 available from National Cancer Institute, Bethesda, Maryland) (19, 20). Joinpoint analysis is a statistical software that allows modeling of trends in data by identifying specific years, or joinpoints, during which significant changes to the data trends occurred. Due to the inclusion of only one age group, infants <1 year of age, CMR was utilized to create the Joinpoint models. The Monte Carlo permutation test was used to calculate the annual percentage change (APC) and average annual percentage change (AAPC) between joinpoints (21). These changes used a 95% confidence interval (CI) and were considered significant if the 95% CI excluded 0 (denoted as asterisks, ‘*’, within the results section).

## 3 Results

### 3.1 Overall

From 1999 to 2023, there were a total of 12,481 deaths of infants <1 year old related to NEC in the US.

**Figure 1:**
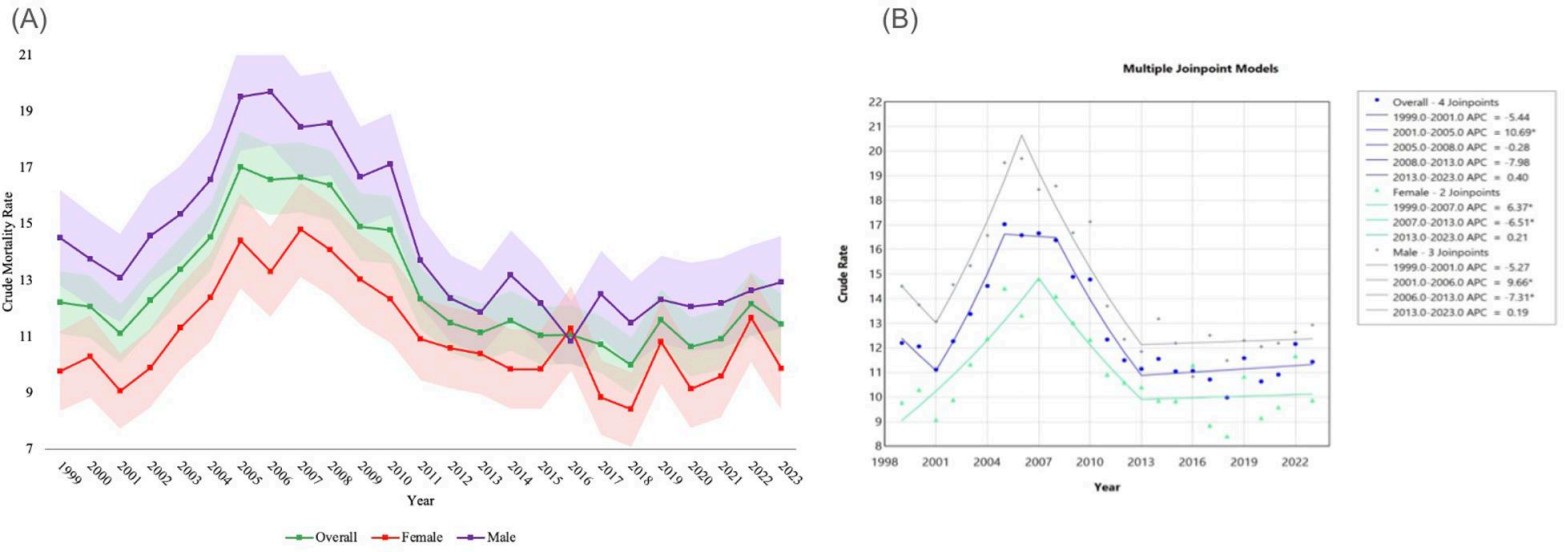
**(A)** NEC-related crude mortality and **(B)** joinpoint model of APC data, overall and stratified by Sex. Legend: Figure 1 describes changes in mortality overall and by sex throughout the study period. NEC-related crude mortality rate is described per 100,000 people overall and stratified by sex between 1999 and 2023 with shading representing confidence intervals. **(A)** Joinpoint models of mortality between 1999 and 2023 are shown in **(B)** Individual regression lines are provided between joinpoints. “*” Indicates the APC is statistically significant.

The overall CMR decreased slightly from 12.20 (95% CI 11.09 to 13.31) in 1999 to 11.43 (95% CI 10.33 to 12.53) in 2023, but ultimately experienced several fluctuations between these time periods. From 1999 to 2001, NEC-related mortality rates demonstrated an APC of −5.44 (95% CI −10.98 to 3.19), followed by a significant increase in associated mortality from 2001 to 2005 to 10.69* (95% CI 7.23 to 16.38). Following this sharp rise, between 2005 and 2008, APC sat at −0.28 (95% CI −8.25 to 4.02). Between 2008 and 2013, APC was −7.98 (95% CI −12.66 to 3.55), and between 2013 and 2023, it was 0.40 (95% CI −2.75 to 2.97). In general, the AAPC for overall deaths related to NEC was −0.37 (95% CI −0.80 to 0.20) reflecting a generally stable mortality rate over the study period. These findings are visualized in Figure 1 and fully displayed in Table 1.

**Table 1:** Overall and stratified by Sex, NEC crude mortality rate per 100,000 people, 1999–2023.

	Crude Mortality Rate (per 100,000)		
Year	Overall	Female	Male
1999	12.20	9.77	14.51
2000	12.06	10.29	13.75
2001	11.11	9.07	13.07
2002	12.27	9.88	14.57
2003	13.38	11.32	15.34
2004	14.52	12.38	16.57
2005	17.03	14.41	19.53
2006	16.58	13.31	19.70
2007	16.66	14.80	18.44
2008	16.38	14.09	18.58
2009	14.89	13.02	16.68
2010	14.78	12.33	17.13
2011	12.34	10.91	13.70
2012	11.49	10.59	12.35
2013	11.14	10.39	11.85
2014	11.55	9.84	13.18
2015	11.04	9.83	12.19
2016	11.06	11.29	10.83
2017	10.71	8.84	12.51
2018	9.98	8.41	11.48
2019	11.58	10.82	12.30
2020	10.63	9.14	12.05
2021	10.91	9.58	12.19
2022	12.16	11.67	12.64
2023	11.43	9.86	12.93
**Number of Joinpoints (Years of Joinpoints)**	4 (2001, 2005, 2008, 2013)	2 (2007, 2013)	3 (2001, 2006, 2013)
**APC Segment-1 (95% CI)**	−5.44 (−10.98 to 3.19)	6.37* (3.87 to 10.10)	−5.27 (−11.24 to 4.02)
**APC Segment-2 (95% CI)**	10.69* (7.23 to 16.38)	−6.51* (−13.80 to −3.05)	9.66* (6.09 to 15.22)
**APC Segment-3 (95% CI)**	−0.28 (−8.25 to 4.02)	0.21 (−1.67 to 6.82)	−7.31* (−10.27 to −5.35)
**APC Segment-4 (95% CI)**	−7.98 (−12.66 to 3.55)		0.19 (−1.03 to 1.75)
**APC Segment-5 (95% CI)**	0.40 (−2.75 to 2.97)		
**Average APC (AAPC) (95% CI)**	−0.37 (−0.80 to 0.20)	0.47 (−0.18 to 1.30)	−0.66* (−1.16 to −0.12)

*Indicates the APC is statistically significant.

### 3.2 Sex

From 1999 to 2023, 5,292 (42.40%) deaths were related to NEC in females and 7,189 (57.60%) deaths in males within the US. The CMR for females negligibly increased from 9.77 (95% CI 8.35 to 11.20) in 1999 to 9.86 (95% CI 8.41 to 11.32) in 2023, reflected by an AAPC of 0.47 (95% CI −0.18 to 1.30). From 1999 to 2007, the APC for female infant deaths related to NEC was 6.37* (95% CI 3.87 to 10.10) followed by a significant decrease from 2007 to 2013 to −6.51* (95% CI −13.80 to −3.05), ending with an APC of 0.21 (95% CI −1.67 to 6.82) from 2013 to 2023.

The CMR for males negligibly decreased from 14.51 (95% CI 12.82 to 16.20) in 1999 to 12.93 (95% CI 11.29 to 14.56) in 2023, but during this time, AAPC was significant at −0.66* (95% CI −1.16 to −0.12)). For males, the APC from 1999 to 2001 was −5.27 (95% CI −11.24 to 4.02), followed by a significant increase to 9.66* (95% CI 6.09 to 15.22) from 2001 to 2006 and a significant decrease of −7.31* (95% CI −10.27 to −5.35) from 2006 to 2013, before ending with an APC of 0.19 (95% CI −1.03 to 1.75) from 2013 to 2023.

Throughout the study, the CMR for male deaths related to NEC was higher than females with the exception of 2016, where male CMR was 10.83 and female CMR was 11.29. The above findings are also visualized on Figure 1 and fully displayed in Supplementary Table S1.

### 3.3 Race and ethnicity

From 1999 to 2023, NEC was implicated in 4,704 deaths in the NH White population, 4,467 deaths in the NH Black or African American population, and 2,630 deaths in the Hispanic or Latino population. NH Black or African American patients consistently had the highest CMR throughout the study period, with a CMR of 33.94 (95% CI 29.15 to 38.72) in 1999 and decreased to 24.81 (95% CI 20.53 to 29.10) in 2023 with a significant AAPC of −1.49* (95% CI −2.03 to −0.76). The APC was also significant, at −12.07* (95% CI −18.67 to −0.46) from 1999 to 2001, after which time it increased to 11.17* (95% CI 7.37 to 18.63) from 2001 to 2006. Between 2006 and 2012, APC decreased again to −9.03* (95% CI −14.28 to −6.16), before ending with an APC of −0.59 (95% CI −1.99 to 1.46) from 2012 to the end of the study.

**Figure 2:**
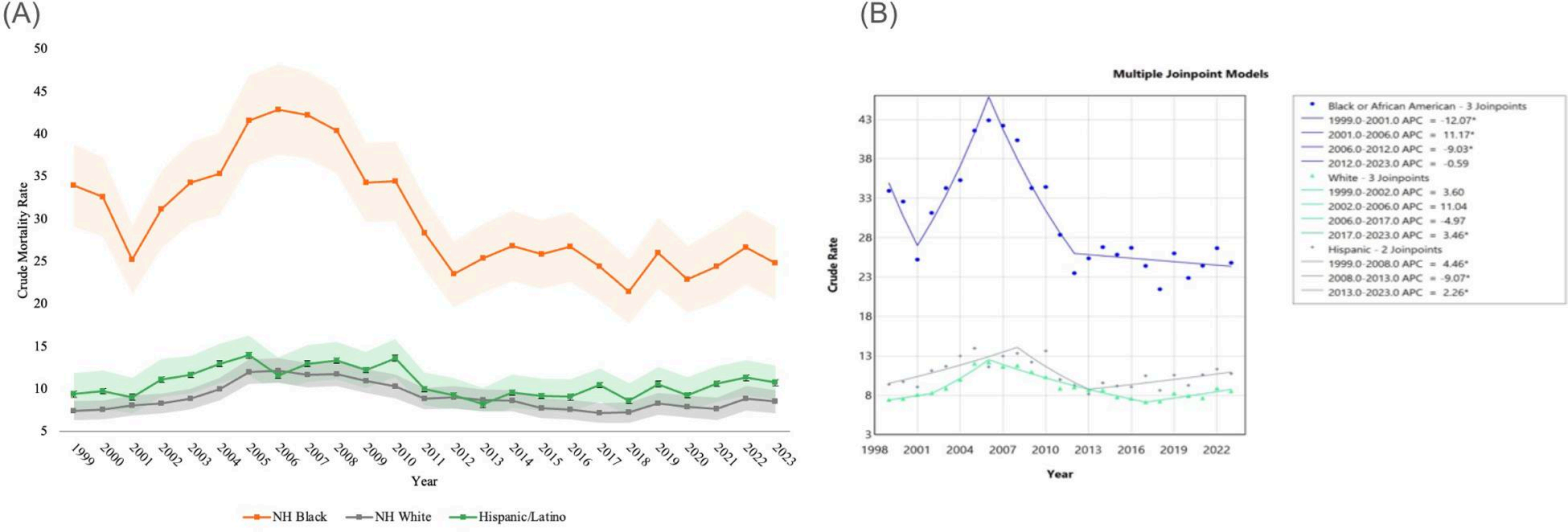
**(A)** NEC-related crude mortality and **(B)** joinpoint model of APC data, stratified by race. Legend: Figure 2 describes changes in mortality by race throughout the study period. NEC-related crude mortality rate is described per 100,000 people, stratified by race, between 1999 and 2023, with shading representing confidence intervals. **(A)** Joinpoint models of mortality between 1999 and 2023 are shown in **(B)** Individual regression lines are provided between joinpoints. “*” Indicates the APC is statistically significant.

CMR related to NEC in the NH White population was 7.44 (95% CI 6.32 to 8.56) in 1999, which negligibly increased to 8.51 (95% CI 7.13 to 9.90) in 2023. Over the study period, AAPC was 0.70* (95% CI 0.27 to 1.22). Between 1999 and 2002, APC was 3.60 (95% CI −2.53 to 9.11). APC then sat at 11.04 (95% CI −5.93 to 14.83) between 2002 and 2006, before decreasing to −4.97 (95% CI −6.82 to 0.93) between 2006 and 2017. APC was then 3.46* (95% CI 0.83 to 7.14) between 2017 through 2023 (Figure 2, Table 2).

**Table 2:** NEC crude mortality rate per 100,000 people, stratified by race, 1999–2023.

	Crude Mortality Rate (per 100,000)		
Year	NH Black	NH White	Hispanic/Latino
1999	33.94	7.44	9.39
2000	32.59	7.55	9.73
2001	25.22	8.07	9.06
2002	31.14	8.30	11.12
2003	34.29	8.84	11.67
2004	35.30	9.99	12.99
2005	41.58	12.02	13.96
2006	42.89	12.18	11.60
2007	42.22	11.63	12.98
2008	40.36	11.77	13.33
2009	34.29	10.96	12.21
2010	34.43	10.32	13.63
2011	28.37	8.85	10.00
2012	23.50	9.03	9.27
2013	25.38	8.67	8.18
2014	26.80	8.63	9.57
2015	25.85	7.75	9.18
2016	26.71	7.59	9.07
2017	24.42	7.15	10.48
2018	21.46	7.22	8.63
2019	26.01	8.26	10.57
2020	22.89	7.93	9.27
2021	24.44	7.64	10.61
2022	26.67	8.90	11.32
2023	24.81	8.51	10.76
**Number of Joinpoints (Years of Joinpoints)**	3 (2001, 2006, 2012)	3 (2002, 2006, 2017)	2 (2008, 2013)
**APC Segment-1 (95% CI)**	−12.07* (−18.67 to −0.46)	3.60 (−2.53 to 9.11)	4.46* (2.36 to 8.17)
**APC Segment-2 (95% CI)**	11.17* (7.37 to 18.63)	11.04 (−5.93 to 14.83)	−9.07* (−16.91 to −4.18)
**APC Segment-3 (95% CI)**	−9.03* (−14.28 to −6.16)	−4.97 (−6.82 to 0.93)	2.26* (0.38 to 6.22)
**APC Segment-4 (95% CI)**	−0.59 (−1.99 to 1.46)	3.4609* (0.83 to 7.14)	
**Average APC (AAPC) (95% CI)**	−1.49* (−2.03 to −0.76)	0.70* (0.27 to 1.22)	0.59 (−0.03 to 1.43)

*Indicates the APC is statistically significant.

Comparatively, in the Hispanic or Latino population, the CMR was 9.39 (95% CI 7.34 to 11.85) in 1999 and negligibly increased to 10.76 (95% CI 8.73 to 12.79) in 2023. AAPC was 0.59 (95% CI −0.03 to 1.43) over the study period (Figure 2, Table 2). More specifically, 1999 to 2008 saw an APC of 4.46* (95% CI 2.36 to 8.17). Subsequently, APC decreased to −9.07* (95% CI −16.91 to −4.18) until 2013, before increasing to 2.26* (95% CI 0.38 to 6.22) through the remainder of the study period Trends in mortality data by race are reflected in Figure 2 and Table 2.

### 3.4 Census region

**Figure 3:**
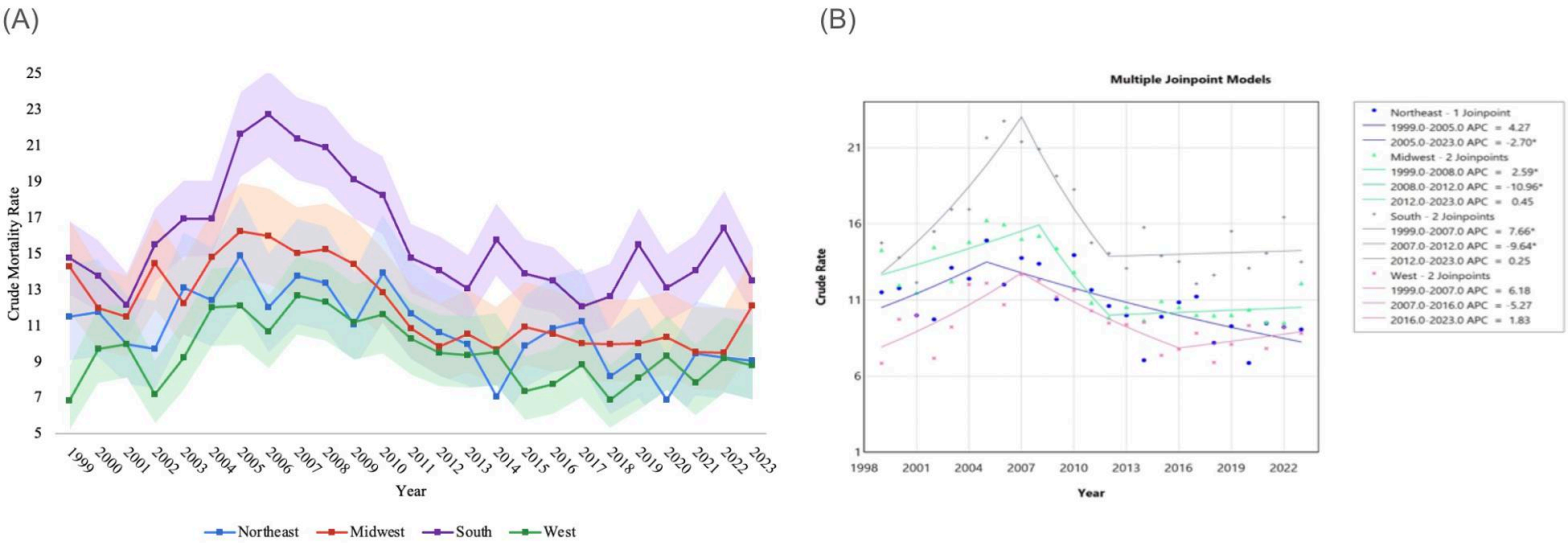
**(A)** NEC-related crude mortality and **(B)** joinpoint model of APC data, stratified by census region. Legend: Figure 3 describes changes in mortality by census region throughout the study period. NEC-related crude mortality rate is described per 100,000 people, stratified by census region, between 1999 and 2023, with shading representing confidence intervals. **(A)** Joinpoint models of mortality between 1999 and 2023 are shown in **(B)** Individual regression lines are provided between joinpoints. “*” Indicates the APC is statistically significant.

Throughout the study period, there were 5,931 deaths related to NEC in the South, 2,282 deaths in the West, 2,536 deaths in the Midwest, and 1,732 deaths in the Northeast. CMR varied across the years throughout the study period. CMR related to NEC remained the highest in the South throughout the study, but trended downward from 14.75 (95% CI 12.72 to 16.78) in 1999 to 13.50 (95% CI 11.63 to 15.37) in 2023, with a negligible AAPC of 0.46 (95% CI −0.29 to 1.53). The APC was 7.66* (95% CI 4.79 to 12.23) from 1999 to 2007, followed by a sharp decrease to −9.64* (95% CI −17.68 to −4.43) from 2007 to 2012, ending at 0.25 (95% CI −1.76 to 6.81) from 2012 to 2023 (Table 3, Figure 3).

**Table 3:** NEC crude mortality rate per 100,000 people, stratified by census region, 1999–2023.

	Crude Mortality Rate (per 100,000)			
Year	Northeast	Midwest	South	West
1999	11.51	14.29	14.75	6.85
2000	11.78	11.98	13.78	9.73
2001	9.99	11.50	12.15	9.99
2002	9.73	14.47	15.50	7.18
2003	13.12	12.23	16.95	9.23
2004	12.40	14.82	16.95	12.02
2005	14.91	16.24	21.65	12.11
2006	12.02	15.97	22.75	10.69
2007	13.76	15.01	21.39	12.68
2008	13.38	15.23	20.91	12.31
2009	11.06	14.40	19.14	11.20
2010	13.95	12.83	18.26	11.64
2011	11.66	10.83	14.76	10.29
2012	10.61	9.84	14.06	9.48
2013	9.99	10.54	13.08	9.38
2014	7.05	9.67	15.77	9.54
2015	9.90	10.93	13.90	7.37
2016	10.85	10.52	13.52	7.77
2017	11.23	10.01	12.07	8.82
2018	8.19	9.98	12.64	6.90
2019	9.28	10.01	15.52	8.09
2020	6.87	10.37	13.09	9.32
2021	9.47	9.55	14.07	7.82
2022	9.22	9.51	16.44	9.21
2023	9.07	12.11	13.50	8.81
**Number of Joinpoints (Years of Joinpoints)**	1 (2005)	2 (2008, 2012)	2 (2007, 2012)	2 (2007, 2016)
**APC Segment-1 (95% CI)**	4.27 (−0.50 to 23.30	2.59* (0.82 to 5.62)	7.66* (4.79 to 12.23)	6.18 (−6.38 to 15.67)
**APC Segment-2 (95% CI)**	−2.70* (−6.50 to −1.73)	−10.96* (−17.20 to −4.69)	−9.64* (−17.68 to −4.43)	−5.27 (−16.59 to 16.92)
**APC Segment-3 (95% CI)**		0.45 (−1.22 to 4.61)	0.25 (−1.76 to 6.81)	1.83 (−3.58 to 19.11)
**Average APC (AAPC) (95% CI)**	−1.00 (−2.32 to 0.21)	−0.77* (−1.37 to −0.01)	0.46 (−0.29 to 1.53)	0.50 (−1.08 to 1.97)

*Indicates the APC is statistically significant.

In the West, CMR in this region negligibly trended upwards from 6.85 (95% CI 5.25 to 8.78) in 1999 to 8.81 (95% CI 6.92 to 11.06) in 2023. In the West, AAPC was 0.50 (95% CI −1.08 to 1.97). Between 1999 and 2007, APC was 6.18 (96% CI −6.38 to 15.67), and between 2007 and 2016, APC was −5.27 (95% CI −16.59 to 16.92). Subsequently, APC between 2016 and 2023 negligibly increased again to 1.83 (95% CI −3.58 to 19.11) between 2016 and 2023 (Table 3, Figure 3).

The second highest CMR was recorded in the Midwest. CMR trended decreased from 14.29 (95% CI 11.75 to 16.83) in 1999 to 12.11 (95% CI 9.74 to 14.89) in 2023, with a significant AAPC of −0.77* (95% CI −1.37 to −0.01). In the Midwest, APC decreased from 2.59* (95% CI 0.82 to 5.62) between 1999 and 2008 to −10.96* (95% CI −17.20 to −4.69) between 2008 and 2012. Through the remainder of the study period from 2012 until 2023, APC was not significant at 0.45 (95% CI −1.22 to 4.61) (Table 3, Figure 3).

In the Northeast, CMR went from 11.51 (95% CI 9.07 to 14.40) in 1999 to 9.07 (95% CI 6.80 to 11.87) in 2023, with an AAPC of −1.00 (95% CI −2.32 to 0.21). Between 1999 and 2005, APC was 4.27 (95% CI −0.50 to 23.30). Subsequently, APC decreased to −2.70* (95% CI −6.50 to −1.73) between 2005 and 2023. Trends in mortality by census region are represented in Figure 3 and Table 3 below.

### 3.5 Urbanization Status

**Figure 4:**
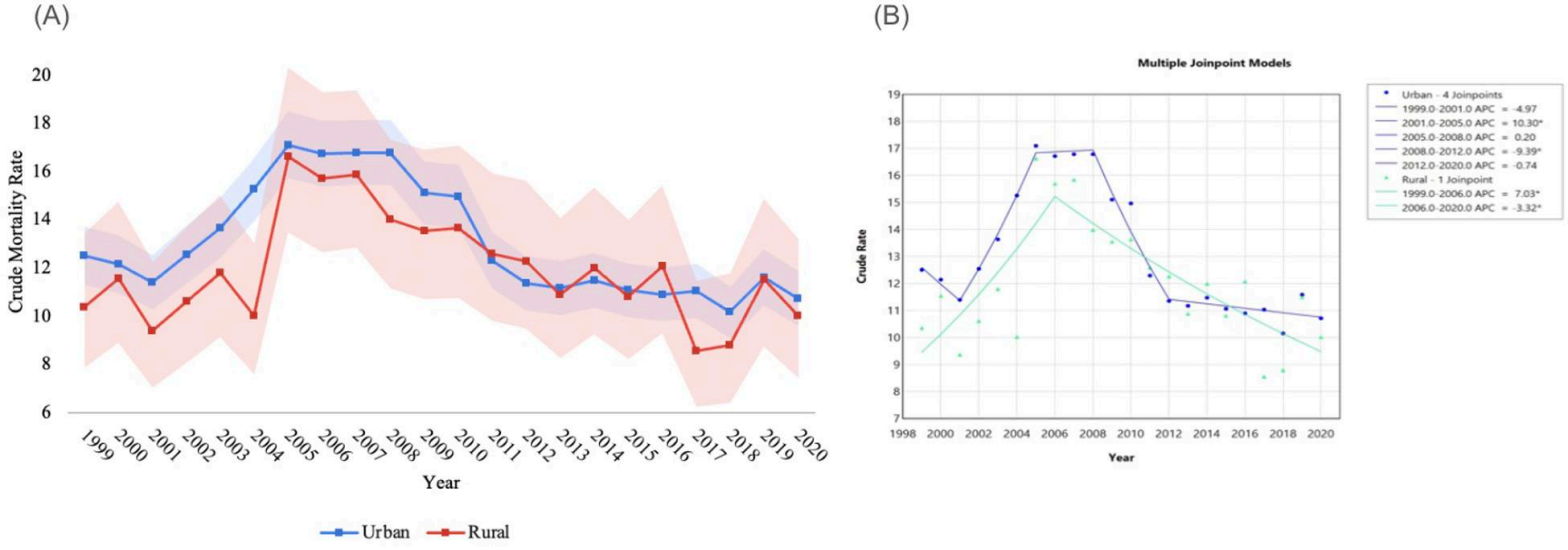
**(A)** NEC-related crude mortality and **(B)** joinpoint model of APC data, stratified by urbanization status. Legend: Figure 4 describes changes in mortality by urbanization status throughout the study period. NEC-related crude mortality rate is described per 100,000 people, stratified by urbanization status, between 1999 and 2020, with shading representing confidence intervals. **(A)** Joinpoint models of mortality between 1999 and 2020 are shown in **(B)** Individual regression lines are provided between joinpoints. “*” Indicates the APC is statistically significant.

NEC was related to a total of 9,775 deaths in urban areas and 1,452 deaths in rural areas. CMR varied among rural and urban areas throughout the study period. In both rural and urban areas, mortality initially increased, before decreasing towards the end of the study period. In rural areas, CMR negligibly trended down overall from 10.36 (95% CI 7.85 to 13.43) in 1999 to 10.02 (95% CI 7.43 to 13.21) in 2020. Between 1999 and 2006, APC was 7.03* (95% CI 2.95 to 17.34), after which it decreased to −3.32* (95% CI −5.67 to −1.91) between 2006 and 2020. Amongst the rural population, AAPC was −0.75* (95% CI −1.12 to −0.37) during the study period (Figure 4, Table 4).

**Table 4:** NEC crude mortality rate per 100,000 people, stratified by urbanization status, 1999–2020.

Year	Urban	Rural
1999	12.51	10.36
2000	12.15	11.55
2001	11.40	9.37
2002	12.55	10.62
2003	13.64	11.80
2004	15.26	10.03
2005	17.10	16.63
2006	16.72	15.70
2007	16.79	15.85
2008	16.79	13.99
2009	15.11	13.55
2010	14.97	13.64
2011	12.30	12.59
2012	11.36	12.27
2013	11.18	10.89
2014	11.48	12.00
2015	11.07	10.81
2016	10.90	12.08
2017	11.04	8.56
2018	10.16	8.80
2019	11.59	11.50
2020	10.72	10.02
**Number of Joinpoints (Years of Joinpoints)**	4 (2001, 2005, 2008, 2012)	1 (2006)
**APC Segment-1 (95% CI)**	−4.97 (−9.16 to 0.47)	7.03* (2.95 to 17.34)
**APC Segment-2 (95% CI)**	10.30* (8.38 to 14.00)	−3.32* (−5.67 to −1.91)
**APC Segment-3 (95% CI)**	0.20 (−6.20 to 5.13)	
**APC Segment-4 (95% CI)**	−9.39* (−12.23 to −6.21)	
**APC Segment-5 (95%CI)**	−0.74 (−1.78 to 0.43)	
**Average APC (AAPC) (95% CI)**	−0.75* (−1.12 to −0.37)	0.01 (−1.23 to 1.52)

*Indicates the APC is statistically significant.

In urban areas, as well, CMR decreased negligibly from 12.51 (95% CI 11.29 to 13.73) in 1999 to 10.72 (95% CI 9.60 to 11.85) in 2020. In these areas, between 1999 and 2001, APC was −4.97 (95% CI −9.16 to 0.47). Between 2001 and 2005, APC increased significantly to 10.30* (95% CI 8.38 to 14.00). Then, from 2005 to 2008, APC sat at to 0.20 (95% CI −6.20 to 5.13), before decreasing significantly to −9.39* (95% CI_−12.23 to −6.21) between 2008 and 2012. Between 2012 and 2020, APC was −0.74 (95% CI −1.78 to 0.43). AAPC was 0.01 (95% CI −1.23 to 1.52) in urban areas over the study period. Trends in mortality across urban and rural areas is described in Figure 4 and Table 4 below.

## 4 Discussion

### 4.1 Discussion and analysis

This study revealed several important trends regarding NEC-related infant mortality from 1999 to 2023; although overall NEC-related CMR has decreased since 2005, there has been negligible improvement in recent years. When stratifying by race, the NH Black or African American population experienced a significantly higher CMR compared to the NH White and Hispanic or Latino population. When stratifying by sex, male infants had a higher CMR related to NEC compared to females. Finally, we noted several regional trends of interest; specifically, the South had the highest CMR throughout the entire study and rural areas had a much higher variability in the 95% CI.

The overall trends observed with NEC-related CMR agree with previous literature and provide updated information for recent years (14). Several factors may contribute to these observed trends. First, the significant increase in NEC-related mortality from 2001 to 2005 may be attributed to the continued improvement in survival of infants of VLBW and early gestational age; as the proportion of premature and VLBW infants increase, so does the rate of NEC (2, 6, 22). The decrease in NEC-related mortality between 2005 and 2013 could be attributed to improvements in diagnostic and management methods for NEC. More recently, however, overall NEC-related CMR has remained stable despite some minor fluctuations. This trend may be attributable to the nonspecific symptoms and laboratory values seen in infants with NEC. Diagnosis requires both identification of symptoms and specific abdominal imaging findings, including pneumatosis intestinalis (23). Even when NEC is properly diagnosed, treatment is limited to supportive care and, when severe, surgical management and continued radiologic evaluation may be necessary (23). As such, diagnostic difficulty and intensive management may be contributing factors to the lack of significant improvement in NEC-related CMR within recent years. These findings emphasize the importance of continued improvement and development of more sensitive and specific diagnostic criteria and management strategies for NEC.

Interestingly, this study found that, with the exception of 2016, male infants generally had a higher NEC-related CMR than females, with a statistically significant difference noted across over half the study period. This reflects a trend that differs from prior studies that found little statistical significance in CMR differences between sexes (23). Through the CDC WONDER database alone, specific reasoning for the higher incidence of CMR for male infants during certain years cannot be determined. However, possible explanations include worsened antioxidant function and poorer immune response in males that may predispose them to higher NEC-related CMR. As such, future study into this is warranted (24).

NH Black or African American infants consistently had the highest NEC-related CMR throughout the study which corroborates previous studies (12–14). Despite limitations to the nature of CDC WONDER’s data and joinpoint analysis, numerous social determinants of health–including economic stability, education access, healthcare access, environment, and social context–have been shown to contribute to the health disparities between the NH Black or African American population and NH White population (25). These same factors may complicate access to prenatal care for conditions that predispose infants to VLBW or prematurity. Given the highly involved process required for diagnosis and management of NEC, any barriers to healthcare may predispose NH Black or African American infants to a higher CMR related to NEC. Other studies have speculated the impact of maternal stress and breastfeeding practices on NEC-related mortality (12). These variations highlight the complicated etiology and multifactorial nature of NEC. In addition, although the disparity between NEC-related CMR has improved from 2006 to 2012 as previously mentioned in other studies, this study’s data shows that this disparity has continued to persist in the past decade without significant improvement since 2012 (14). These findings emphasize the necessity of studying the social determinants of health and the structural and systemic barriers that contribute to them.

When stratifying by census region, the South consistently had a higher CMR than the other three regions throughout the study. This difference in CMR between the South and other regions has slightly decreased from 2011 to the end of the study. The potential reason for increased mortality in the South is multifactorial. Existing literature notes that the Southeast US has the highest number of NH Black or African American preterm births across the US, and, regardless of race, VLBW infants in the South had the least human milk consumption at time of discharge (26). Moreover, they note that Black or African American infants were even less likely to have been provided human milk upon discharge (26). Both race and the overall decreased discharge on human milk can increase the risk of NEC. Preterm birth is likely the major contributor to NEC, and the lack of protective factors in these patients may explain the increased mortality from NEC (26).

When stratifying by urbanization status, rural areas tended to have a much wider variation in their 95% CI. This variability is likely due to higher comparative sample size in the urban population. Because of this variation, this study did not find a significant difference in NEC related mortality rate between urban and rural areas. In rural areas, mothers and their neonates have decreased access to care, leading to an overall elevated risk of complications for both (27). In fact, preterm births were notably more frequent in rural areas (27). However, infants born in rural areas were less likely to be admitted to the NICU and were more likely to be large for gestational age compared to their urban counterparts (27). This, in addition to the overall higher concentration of patients localized in urban centers, may contribute to the complicated nature of urban and rural area disparities (28). Despite this, disparities persist in urban-rural mortality, offering a potential explanation for the oscillations in the trends between the two cohorts.

### 4.2 Limitations

This study is not without limitations. Firstly, our study is limited to information being provided by the CDC WONDER database, which utilizes information included on patient death certificates (29). As such, inclusion within this study requires NEC to be mentioned as a contributing cause of mortality on these documents. Moreover, we are unable to further stratify patients by other contributors to mortality. While our study includes all patients with a listed diagnosis of NEC contributing to mortality, other comorbid conditions may confound trends in mortality that we observe. The joinpoint analysis additionally does not allow for the adjustment of covariates, limiting this study’s interpretation to associations and disparities between populations only. Speculations for said associations and disparities are listed in the discussion section, but additional studies would have to be completed to confirm said suspicions. In addition, our study does not include information on treatment protocols each patient received, so we are unable to determine how the type of intervention and time to intervention impacted survival in these patients. In addition, this study utilizes CMR to describe mortality rate. However, the use of CMR alone cannot independently account for how ecological shifts, such as changes in population characteristics over time, account for changes in mortality trends. As such, this study highlights important trends useful for guiding strategies to improve NEC outcomes, but future study using standardized analysis will be beneficial to build on this analysis. CMR was further calculated based on the <1 year of age category in the “Ten-Year Age Groups,” which uses the total population of this age group as opposed to live births (infant mortality rate). Finally, for cohorts within certain demographic variables in which there was a death count of less than 20, rates were marked as unreliable, limiting further assessment and analysis of mortality within these groups.

## 5 Conclusion

This study highlights the disproportionate mortality burden experienced by certain groups, including males, NH Black or African American patients, and patients living in the Southern US, in order to serve as a guide for future research into improved management of NEC. In addition to understanding social and structural factors that may predispose infants to NEC, further studies should aim to better understand biologic, hormonal, and genetic factors that increase an infant’s risk for NEC. Moreover, given that numerous diagnostic factors central to NEC remain nonspecific findings, further study should prioritize understanding how the identification of infants at increased risk and improved monitoring can improve outcomes in these patients.

## Data Availability

The original contributions presented in the study are included in the article/Supplementary Material, further inquiries can be directed to the corresponding author.
